# Systematic Review: Should Routine Resection of the Extrahepatic Bile Duct Be Performed in Gallbladder Cancer?

**DOI:** 10.4103/1319-3767.65184

**Published:** 2010-07

**Authors:** Parul J. Shukla, Savio G. Barreto

**Affiliations:** Department of Gastrointestinal Surgical Oncology, Tata Memorial Hospital, Mumbai, India; 1 Department of General and Digestive Surgery, Flinders Medical Center, Adelaide, Australia

**Keywords:** Aggressive surgery, gallbladder, hepatoduodenal ligament, metastasis

## Abstract

**Background /Aim:**

Complete surgical resection is associated with improved outcomes in gallbladder cancer. Whether the extra-hepatic bile duct (EHBD) should be routinely excised for gallbladder cancer is unclear. Objective: To analyze literature concerning EHBD excision to determine if it is associated with survival advantage and hence can be routinely recommended.

**Materials and Methods:**

A systematic search using Medline, Embase, and Cochrane Central Register of Controlled Trials for the years 1988-2008.

**Results:**

EHBD excision was reported to be performed routinely for T1-4 in some studies, while others reported resection to facilitate lymph node clearance or if the EHBD was grossly involved by disease that remained otherwise resectable. While one study demonstrated 100% survival in T1 disease, other reports do not demonstrate any survival benefit of EHBD excision in T1 disease. Four studies (level IV-V) demonstrated 60% to 90% five-year survival for routine excision in T2 disease, while three other studies demonstrated no survival advantage but increased morbidity due to the procedure. In T3/4 disease, one study (level IV-V) demonstrated a benefit in T4 disease only, and another study (level IV-V) reported a survival advantage in patients in whom the bile duct was not involved; five other studies showed no impact of routine EHBD excision on survival but reported morbidity following anastomotic leaks.

**Conclusions:**

Available evidence does not support routine resection of EHBD in gallbladder cancer. EHBD excision should be performed in the presence of specific indications, viz., to achieve an R0 resection of the primary tumor and/ or to aid complete lymph node dissection that would compromise the EHBD by devascularization.

It is widely accepted that complete surgical resection offers the best chance of long-term survival in gallbladder cancer.[1–18] However, the words ‘complete,’ ‘radical’ and ‘aggressive’ surgeries have been used interchangeably in literature, rendering the comparison of results of surgical resections difficult. This in turn leads to difficulties in laying down protocols for the surgical management of gallbladder cancer.

While some surgeons routinely recommend the resection of the extra-hepatic bile duct (EHBD) for gallbladder cancer, the possibility of post-EHBD-resection complications and the lack of a survival advantage lead us to the question, “Does the EHBD need to be routinely resected in gallbladder cancer?”

While the patterns of spread of gallbladder cancer have been presented by various authors,[13,19,20] the underlying etiopathogenesis has been rarely considered.[20] Moreover, the rationale behind performing an EHBD resection needs to be understood. We thus undertook this review of literature with an aim to answer the following questions:

- How is the EHBD involved in gallbladder cancer?
- What is the justification of an EHBD resection?
- Should the bile duct be resected in all stages of gallbladder cancer?

## MATERIALS AND METHODS

An extensive and systematic Medline and Embase search was performed to identify the existing literature on resection of the EHBD in gallbladder cancer. The search terms used included ‘gallbladder cancer,’ ‘extra-hepatic bile duct,’ ‘resection,’ ‘hepatoduodenal ligament,’ ‘radical,’ ‘aggressive,’ ‘surgery,’ ‘anastomotic leak.’ Using the above search terms yielded a total of 981 publications published in the last 30 years. These publications were then analyzed specifically looking at those papers which addressed the resection of the extra-hepatic bile duct in gallbladder cancer. Of the 51 papers thus retrieved and referenced in the current review, only 15 that actually looked at the impact of extra-hepatic bile duct resection on outcomes of gallbladder cancer were analyzed.

## RESULTS

### Involvement of the EHBD in gallbladder cancer

While considering the development of gallbladder cancer, it is important to realize that there are differences in the pathophysiology of gallbladder cancer in different parts of the world.[21] The basic differences lie in the fact that the etiology, viz., gallstones or the anomalous pancreaticobiliary duct junction (APBDJ), results in certain gene mutations (K-ras and p53)[22] and consequent patterns of disease progression to the final malignant state. Gallstones have been noted to be associated with an increased risk of p53 mutations and the consequent risk of metaplasia and onward progression to dysplasia and invasive cancer. Patients with APBDJ, on the other hand, have been noted to have an increased risk of K-ras mutations, and the pattern of progression here is hyperplasia developing into papillary tumors and finally invasive cancer.

The presence of cancer in the EHBD in a patient with gallbladder cancer can arise from broadly two clinical scenarios: cancer of the gallbladder due to causes such as gallstones, preexisting porcelain gallbladder or gallbladder polyps following which the EHBD may be involved by three of the four patterns described by Shimizu *et al*.,[13] viz., type I (direct spread from the primary tumor), type II (continuous intramural spread along the cystic duct to the EHBD) and type IV (permeation of tumor cells from metastatic lymph nodes in the hepatoduodenal ligament).

The second scenario includes the involvement of the EHBD noncontiguous with the gallbladder tumor or the type III tumor described by Shimizu *et al*.[13] The basis for this lies in the concept of field cancerization[20] — the entire biliary tree is at a risk for developing a malignancy due to exposure to a potentially carcinogenic process or substance. In such cases, the method of involvement of the EHBD can be divided into retrograde — secondary to an APBDJ with the consequent exposure of the biliary tree to the refluxing mixture of pancreatic and biliary juices[23,24]; or antegrade (gallbladder cancer along with a synchronous/ metachronous malignancy in the EHBD distal to the attachment of the cystic duct) — as seen in patients with gallstones but in the absence of APBDJ. A hypothetical explanation for the latter scenario could possibly be alterations in the bile acid content after its entry into the gallbladder, which leads to the exposure of the gallbladder and the portion of the EHBD distal to the insertion of the cystic duct to the potentially harmful effects of the altered bile. Srivastava *et al*.[25] recently demonstrated differences in cholesterol, calcium and magnesium composition in gallstones in patients with gallbladder cancer and chronic cholecystitis using proton nuclear magnetic resonance spectroscopy. Whether such changes in the inorganic composition of bile and gallstones occur within the gallbladder exposing the EHBD to the altered bile and hence the possibility of developing malignant change, is yet to be determined.

However, despite the above-described pathways, the actual incidence of synchronous lesions in the EHBD in a patient with gallbladder cancer is small. In series from Japan, where APBDJ as a cause for gallbladder cancer is more common, the incidence of synchronous tumors in the EHBD and the gallbladder has been reported to be only 5% to 7.4%.[26–28]

### Routine excision of the EHBD

EHBD excision is included as part of a radical resection for all stages of gallbladder cancer by Japanese surgeons.[5,7,8] The reasons cited for this have been — in early stages, to aid clearance of the hepatoduodenal ligament of lymph nodes and occult cancer cells in the connective tissue[13] ; and in the advanced stages, as part of radical resection of the malignancy to address the issue of perineural invasion.[15] They also believed that radical resections that include the excision of the EHBD, even in T1 tumors, were associated with a survival advantage.[5,8]

Further data supporting the routine resection of the EHBD have been listed below [Table 1][29]

**Table 1:** Levels of evidence of studies supporting routine EHBD resection for gallbladder cancer

Author (Ref)	No. of patients	Conclusions	Level of evidence[29]
Studies supporting routine EHBD excision in T2 disease			
Suzuki *et al*.[30]	20 (T2 disease)	5YSR- 77%	IV - V
	8 / 20 - no EHBD excision	5YSR - 100%	
Shimada *et al*.[31]	41	3YSR	IV - V
	T1 - 4	100%	
	T2 - 21	74.8%	
	T3/4 - 16	6.7%	
Nagakura *et al*.[32]	63	Poor survival in patients with overt and micrometastases to nodes	IV - V
Shirai *et al*.[34]	48	5YSR - 90%	IV - V
Wise *et al*.[36]	5	100% disease free at follow-up ranging from 15 to 83 months	V
Chijiiwa *et al*.[37]	52	5YSR	IV - V
		T1 - 100%	
		T2 - 60.8%	
		T3/4 - 0%	
Studies supporting routine EHBD excision in T3/4 disease			
Todoroki *et al*.[9]	135	5YSR	IV - V
	T1 - 13	100%	
	T2 - 24	70%	
	T3 - 9	19%	
	T4 - 89	5%	
Kosuge *et al*.[38]	55	No difference in survival with or without EHBD excision in stages 1-3 but only for stage 4	IV
Kaneoka *et al*.[39]	59	Benefit of bile duct resection is restricted to patients without bile duct invasion	IV - V

### Routine resection of the EHBD in T2 disease

Suzuki *et al*.[30] suggested that routine resection of the bile duct should be performed in patients with pT2 disease, based on their experience in treating 20 patients. However, in 8 of the 20 patients who had tumors in the fundus and body of the gallbladder, the EHBD was not resected and the median survival in this group was 64 months with no recurrence. Besides, they also had two anastomotic leaks (16.7%). Shimada *et al*.[31] supported the routine excision of the EHBD for T2 disease but not T3/T4, based on their experience with 41 patients in whom they performed a radical resection including excision of the EHBD for all patients with pT2 disease and above. They found that in patients with T2 disease there was a three-year survival rate of 60%; and a five-year survival rate of 49% in those who underwent a curative resection as opposed to 0% three-year survival rate in those who did not undergo a curative resection. Nagakura *et al*.[32] found that nodal micrometastasis and perineural invasion[15] were important determinants of post–radical-resection survival in gallbladder cancer. However, they did find that perineural invasion was uncommon in T1b cancers, which tended to spread locally.[33] They also found that extended resections were significantly associated with improved survival in the 54 patients with T2-4 disease. Based on these findings, they suggested that routine resection of the EHBD is essential in all patients with T2-4 disease.[32] Shirai *et al*.[34,35] recommended routine resection of the EHBD for pT2 lesions and above either as a primary procedure or as part of radical re-resection in the case of incidental gallbladder cancers, based on the long-term survival encountered in these patients as a result of radical resections. Interestingly, in the 6 long-term survivors with pT2 disease,[34] while they performed lymph node dissections and wedge resections of the liver in all the patients, they had actually performed bile duct resections in only 3 (50%) patients. Wise *et al*.[36] suggested that EHBD should be resected as part of a radical resection in all patients with stage 2 or more gallbladder cancer, based on their experience in 5 patients (stage 2=3, stage 3=2) who underwent radical resections and were disease-free at follow-up ranging from 15-83 months. Chijiiwa *et al*.,[37] too, advised EHBD resection as part of radical resection for all patients with stages 1-3 gallbladder cancer, based on the perceived survival advantage. They performed a bile duct resection (either alone or as part of a pancreatoduodenectomy) in 24 of the 52 patients studied and encountered three anastomotic leaks. They found that radical resection did not offer any survival advantage to patients with stage 4 disease.

### Routine resection of the EHBD in T3/T4 disease

Todoroki *et al*.[9] recommend routine EHBD resection as part of curative resection for T2-4 tumors, based on the rationale that once cancer cells breach the serosa, they are likely to involve all structures contiguous with the gallbladder, viz., the cystic duct, the EHBD, the Glisson‘s capsule, the hepatoduodenal ligament, adjacent nervous and lymphatic tissues and the vessels that are covered by the serosa. This has also been confirmed by Shimizu *et al*.[13] Kosuge *et al*.[38] recommended routine resection of the EHBD in stage IV disease, based on their findings of significantly improved survival in patients with stage IV disease with hepatoduodenal ligament invasion who underwent EHBD resection as compared to those who did not. They however also found that in stages lower than IV, survival was unaffected by resection of the EHBD. Aggressive surgical resection including the excision of the EHBD has been shown to lead to a 13.7% five-year survival rate in those patients who underwent a curative resection.[16] This study by Shimizu *et al*.[16] did record a morbidity rate of 48.1% and an in-hospital mortality rate of 11.4%.

Kaneoka *et al*.[39] routinely performed resection of the EHBD for stages II-IV gallbladder cancer. They stratified patients based on the presence/ absence of lymph node and bile duct involvement. By this strategy, they were able to achieve a curative resection in 75% of patients without bile duct involvement but <30% of patients with bile duct involvement. Further, they reported a three-year survival rate of 35.3% to 65.6% in those patients with curative resection and without bile duct involvement and a thee-year survival rate in only 5.9% to 14.3% of patients with bile duct involvement, with or without lymph node involvement. There were no five-year survivors amongst those with bile duct involvement.

### Evidence against routine excision of EHBD

Considerable data has accumulated over the last few years to substantiate the argument that routine excision of the EHBD is not warranted in gallbladder cancer [Table 2]. The two main reasons for this are that it has not provided sufficient evidence to suggest a positive influence on survival[11,31,42–43]; and secondly, the resection of the EHBD along with reconstruction has been linked to an increased risk of early (biliary anastomotic leak and collections) and late sequelae like strictures and attendant cholangitis.[11,31,42–44]

**Table 2:** Stage-wise distribution of studies highlighting the lack of benefit of routine EHBD resection for gallbladder cancer

Study	Stage	Effect on survival	Complication
Chijiiwa *et al*., 2001[11]	T2 N0-2	None	Anastomotic leak
Pawlik *et al*., 2007[41]	*n*=42; T2	None; no effect on number of lymph nodes harvested	Not specifically addressed
Shimada *et al*., 1997[31]	T3/4	None	Anastomotic leak
Bartlett *et al*., 1996[44]	*n*=10; all stages	Not specifically addressed	50%
Kokudo *et al*., 2003[40]	*n*=33; all stages	None	Not specifically addressed
Muratore *et al*., 20001[42]	*n*=33; all stages	None	High morbidity and mortality
Behari *et al*.[43]	*n*=10; all stages	None	Bile leak

*CHD — common hepatic duct

## DISCUSSION

Literature on the role of EHBD resection for gallbladder cancer is confusing and at times contradictory. This probably stems from a number of factors: the most likely being the low number of cases of gallbladder carcinoma and their uneven distribution around the world, the often delayed presentation by patients who attribute the early symptoms to other common benign diseases including gallstones, a possible difference in aggressiveness amongst surgeons while managing such patients, and the possibility of a varied pathogenesis (as highlighted above). Also, the management strategies employed in the treatment of gallbladder cancer center around the T stage. However, the inability to accurately predict this T stage preoperatively leads to further confusion, with some surgeons preferring to do more, rather than less, given the dismal prognosis associated with a non-curative resection. So, is there sufficient evidence to support the routine resection of the bile duct irrespective of the T stage of the disease?

A closer analysis of the studies supporting the routine resection of EHBD for early stages seems to indicate that there have been instances where some groups have chosen to be conservative with regard to the EHBD, and it has not affected survival.

In the case of patients with T1 disease, Shimada *et al*.[31] found that EHBD excision in 4 patients was associated with a 100% five-year survival. While it is widely accepted that a simple cholecystectomy constitutes an oncologically acceptable operation, the debate on the appropriate surgery for T1b tumors continues. Wakai *et al*.[33] found no benefit of a radical resection in patients with T1b tumors. They based their conclusions on the fact that they found no nodal metastasis in 12 out of 25 patients in whom a radical resection was performed. In the author‘s experience,[1] T1b tumors were found to metastasize to lymph nodes and thus routine lymph node dissection along with resection of the gallbladder fossa (liver wedge) is justified in these patients — a view shared by Muratore *et al*.[45] The author’s data also supports that of Wagholikar *et al*.,[47] who found that performance of a simple cholecystectomy in T1b tumors resulted in a five-year survival rate of 68%, with 5 out of the 12 patients studied developing locoregional recurrences.

Suzuki *et al*.[30] noted that 8 (out of 20) patients with gallbladder cancer in whom the tumor was located in the body and fundus, and in whom the EHBD was preserved, did see a 100% five-year survival rate with no recurrences. Similarly, Shirai *et al*.[34] compared radical resection with simple cholecystectomy for T2 disease. In their study, they had actually preserved the EHBD in 50% of their long-term survivors with T2 disease. In the paper by Nagakura *et al*.,[32] the outcome of an extended cholecystectomy was actually compared with the outcomes of hepatectomy and pancreaticoduodenectomy and not with those in patients who had not had bile duct resections. (The authors did mention they had not performed a routine bile duct resection in some patients with early-stage disease.)

Further, the data supporting the role of routine EHBD resection in stages II-IV seems to be contradictory based on the conclusions of Chijiiwa *et al*.[37] and Kosuge *et al*.[38] Studies compiled in Table 2 seem to agree with the findings of Kosuge *et al*.[38] that in early stages, especially T2 disease, the addition of a resection of the EHBD does not offer any survival advantage but only increases postoperative morbidity. However, we should not be quick to question the benefit of, or need for, EHBD resection in all T2 diseases. This is because, in the presence of large lymph nodal disease that may be encountered in T2 tumors, the resection of the EHBD may be necessary to aid clearance of the hepatoduodenal ligament.

Thus, for T2 cancers and even some T3 tumors, in the absence of gross nodal disease, a resection that includes a cholecystectomy with a wedge resection of the gallbladder bed/ segment IVB and V resection, along with a regional lymph nodal dissection, has been shown to constitute a curative surgery.[4,42,46–49]

The role of EHBD resection in the presence of bile duct involvement is also very confounding. However, what seems to emerge is that the involvement of the bile duct in gallbladder cancer is itself a poor prognostic indicator of survival. Sikora *et al*.[14] had previously noted that in the series of patients reported by Shimizu *et al*.,[13] the overall survival in patients with overt or occult involvement of the bile duct was low, thus implying that the EHBD resection was serving more as a staging procedure rather than a curative option. This negative impact of bile duct involvement on survival has also been highlighted by other authors.[31,39,40] Besides, there is also sufficient evidence to suggest that the inability to obtain a curative resection (with negative margins), should preclude further attempts at aggressive resection, as this only increases the likelihood of morbidity without positively influencing survival.

The studies highlighted in Table 2 do not support the idea of routine resection of the EHBD in gallbladder cancer but do provide specific indications for it.

Indications for the resection of the EHBD in all stages of disease include —

1. Tumors involving the EHBD[50] - Preoperatively indicated by the presence of obstructive jaundice, in the absence of distant metastasis
2. Tumors/gross lymph nodal enlargement close to or involving the common hepatic duct[44] or hilum[30]
3. Inflamed or a fatty hepatoduodenal ligament rendering nodal dissection difficult[44]
4. Patients undergoing re-resection (since postoperative inflammation makes differentiation of tumor and scar difficult).[44,50] This indication is optional as in the authors’ own experience, the extent of hepatoduodenal ligament inflammation and fibrosis need not always preclude a complete clearance[1]
5. Positive cystic duct margin on intraoperative frozen section[1]
6. Cystic duct cancers[30,43]
7. Patients with associated APBDJ[40] /choledochal cysts of the EHBD — these patients are at an increased risk of further metachronous malignancies of the biliary tree and should hence undergo EHBD resection at the time of treatment of the gallbladder cancer
8. In case of need for associated vascular resection/ reconstruction[51]

## CONCLUSION

While theoretically there is the possibility of involvement of the EHBD in gallbladder cancers, the synchronous existence of a malignancy in the EHBD and the gallbladder is uncommon. In the absence of convincing data to demonstrate a survival advantage for the routine excision of the EHBD in gallbladder cancer, the morbidity of the procedure needs to be taken into consideration. This is because the most important complication following bile duct resection and reconstruction, is the development of an anastomotic leak with its attendant short-term sequelae of sepsis and peri-operative mortality; also, there are long-term effects like stricture formation and repeated attacks of cholangitis, rendering the patient a “biliary cripple.” Given the biases in different parts of the world with regard to the excision of the EHBD, it seems unlikely that a randomized controlled trial would be undertaken. In the absence of level 1 evidence to support the routine resection of the EHBD in gallbladder cancer, such a resection should be performed only in the presence of the specific indications, viz., to achieve an R0 resection of the primary tumor and/ or to aid the performance of a complete lymph node dissection that would compromise the EHBD due to devascularization.
